# The idiosyncrasy of spatial structure in bacterial competition

**DOI:** 10.1186/s13104-015-1169-x

**Published:** 2015-06-17

**Authors:** Felix J H Hol, Peter Galajda, Rutger G Woolthuis, Cees Dekker, Juan E Keymer

**Affiliations:** Department of Bionanoscience, Kavli Institute of Nanoscience, Delft University of Technology, Lorentzweg 1, 2628 CJ Delft, The Netherlands; Institute of Biophysics, Biological Research Centre of the Hungarian Academy of Sciences, Temesvari krt. 62, Szeged, Hungary; Department of Ecology, Faculty of Biological Sciences, P. Catholic University of Chile, Alameda 340, Santiago, Chile; Institute of Physics, Faculty of Physics, P. Catholic University of Chile, Ave. Vicuña Mackenna 4860, Macul, Santiago, Chile

**Keywords:** Spatial structure, Bacterial competition, Cooperation

## Abstract

**Background:**

The spatial structure of a habitat can have a strong impact on community dynamics. Different experimental approaches exist to explore the effect of spatial structure on bacterial communities. To investigate the effect of ‘space’, a single implementation of spatial structure is often contrasted to bacterial community dynamics in well-mixed cultures. While such comparisons are useful, it is likely that the observed dynamics will be particular to the specific experimental implementation of spatial structure. In order to address this question, we track the community dynamics of a two-strain *Escherichia coli* community in various spatial habitats and relate the observed dynamics to the structure of a habitat.

**Results:**

By tracking the community dynamics of *rpoS* wild-type and mutant *E. coli* in radially expanding colonies on solid and semi-solid agar plates, we find that the mutant strain outcompetes the wild-type on semi-solid agar plates, whereas the two strains coexist on solid agar. We compare these results to previous studies in which the same two strains were shown to coexist in habitats spatially structured by microfabrication, while the mutant outcompeted the wild-type in well-mixed batch cultures. Together, these observations show that different implementations of space may result in qualitatively different community dynamics. Furthermore, we argue that the same competitive outcome (e.g. coexistence) may arise from distinct underlying dynamics in different experimental implementations of spatial structure.

**Conclusions:**

Our observations demonstrate that different experimental implementations of spatial structure may not only lead to quantitatively different communities (changes in the relative abundance of types) but can also lead to qualitatively different outcomes of long-term community dynamics (coexistence versus extinction and loss of biodiversity).

## Background

Ecological as well as physiological interactions between cells are strongly determined by their adjacency relationships. The spatial structure of an environment sets the geometry of cell–cell interactions [1], and community dynamics therefore depend critically on spatial structure [2]. Traditionally, well-mixed flasks have been the method of choice to culture microbial populations. While invaluable for microbiological experiments, this culture method is chemically homogeneous and spatially unstructured, imposing a network of interactions where all cells interact with one another in a similar (mean-field) fashion, thus lacking ecological realism. The importance of spatial structure in governing microbial interactions has long been recognized, and various experimental approaches to implement spatial structure have emerged. Experimental implementations of spatial structure are diverse and range from e.g. the periodic dispersal of unstructured subpopulations [3–5], to radially expanding colonies growing on an agar surface [6–10], and synthetic ecosystems created by microfabrication [11–18]. In contrast to cultures growing in a well-mixed flask, these spatial constructions have in common that cells interact with a limited number of partners over relatively short spatial scales. However, there are important differences between the various implementations of spatial structure, for instance concerning cellular motility and dispersal mode. The particular experimental implementation of spatial structure is thus likely to affect the competitive dynamics of its inhabitants. Yet a systematic study of the same biological community in various spatial systems is currently lacking. In order to investigate whether different implementations of spatial structure result in qualitatively different community dynamics, we study a defined biological community (wild-type and *rpoS* mutant *Escherichia coli* bacteria) in different spatial constructions while keeping parameters like growth medium and temperature constant.

The influence of spatial structure is of particular interest for bacteria that are engaged in a social dilemma, as a multitude of theoretical work suggests that spatial structure can have a profound influence on social interactions [19–27]. Although various theoretical studies suggest that spatial structure stabilizes coexistence in competitive social interactions, in practice, different implementations of spatial structure will induce distinct interaction networks and therefore may give rise to different community dynamics and long-term community structure. We investigate a community consisting of an *E. colirpoS* wild-type (WT) and an *rpoS819* mutant strain. The *rpoS* gene encodes for *E. coli*’s general stress regulator $\sigma ^S$. Activity of $\sigma ^S$ is triggered by nutrient deprivation and various other stress factors. The *rpoS819* allele confers a growth advantage in stationary phase (GASP) phenotype which is characterized by a delayed entry into stationary phase [28–30]. The WT and GASP strain are engaged in a social dilemma concerning resource use and management: in a well-mixed batch culture, WT cells cooperate by collectively going into stationary phase and restrain growth *before* all resources are depleted, ensuring that enough resources remain available to sustain a viable population for long periods. GASP mutants on the other hand, defect on this social contract by ignoring the collective decision to restrict growth, using the scarce resources for growth instead of investing in maintenance [29, 31]. RpoS WT and GASP mutants have previously been shown to interact according to a prisoner’s dilemma game in unstructured cultures (well-mixed flasks) [12, 29]. In the absence of spatial structure, GASP cheaters were found to drive WT cooperators extinct as predicted by theoretical studies of the prisoner’s dilemma. Previous experimental studies of this *E. coli* community furthermore have confirmed that the WT cooperator strain can coexist with the GASP cheater strain in habitats that were spatially structured using microfabrication: Refs. [12, 32, 33] followed the community dynamics of a WT-GASP *E. coli* community in engineered microhabitats and demonstrated that the WT and GASP strain indeed coexist at intermediate frequencies in this type of environments. To investigate whether different implementations of spatial structure indeed lead to different dynamics of the same community, we investigated WT-GASP competition in colonies growing on agar plates.

## Results

**Figure 1:**
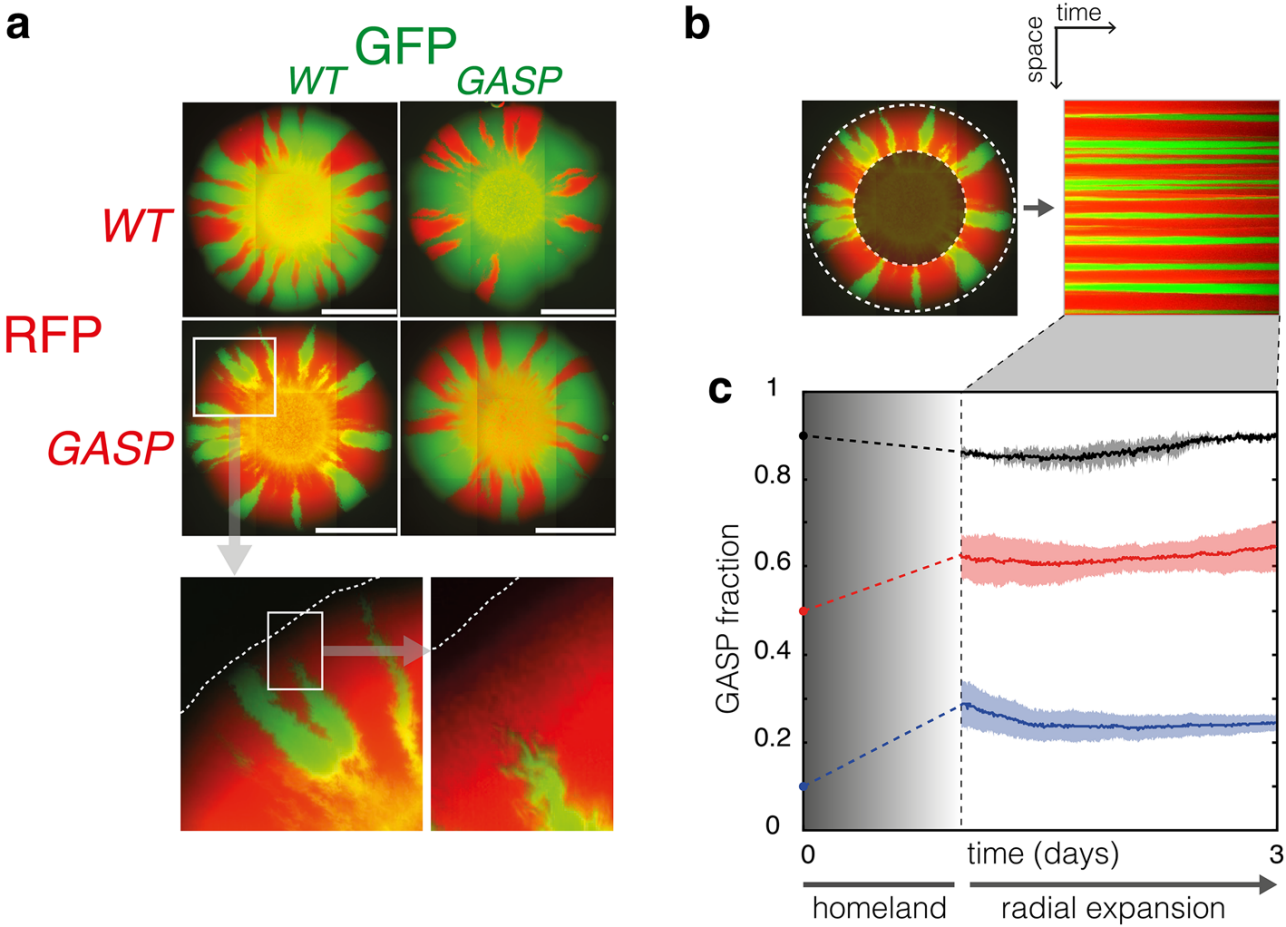
The WT-GASP community on solid agar plates. **a** Colonies, inoculated with 1:1 mixture of WT/GASP bacteria (*left bottom* and* right top*), growing on solid plates show a typical sectoring pattern, as indicated by the* red* or* green* fluorescent proteins they express. Controls, WT/WT (*left top*) and GASP/GASP (*right bottom*) have a* red* fraction of 0.44 ± 0.12 (*n* = 6, mean ± standard deviation) and show that the fluorescent reporters are neutral. All *scale bars* indicate 3 mm. The *bottom panel* shows an enlarged view of the area depicted by the *white square* on the GASP-WT plate, the advancing edge of the colony is represented by a* dashed line*. **b** The area in between the *dashed lines* on the plate image is converted to polar coordinates depicting space vertically and time horizontally. The time evolution of the GASP fraction can be easily obtained from the converted image. **c** Traces showing the GASP fraction during the sectored growth phase.* Solid lines* show the means of GASP fractions for colonies inoculated with GASP fractions of 0.1 (*blue* *n* = 6), 0.5 (*red* *n* = 6), and 0.9 (*black* *n* = 2);* shaded areas* indicate the standard deviation. GASP fractions at* t* = 0 show the inoculated fractions. The GASP fraction increases during formation of the homeland, while the GASP fraction remains constant in the subsequent radial expansion which typically starts after ~1 day.

The use of radially expanding colonies growing on agar plates has recently attracted interest as an experimental system to study microbial competition in space (e.g. Refs. [6–10, 34]). In this approach the surface of a solid agar plate is inoculated with a droplet consisting of a multi-strain mixture, and a sectored colony develops (see Figure 1a). Community development in this setting occurs in two phases: initially, growth occurs in distinct microcolonies that arise from individual cells that were inoculated on the plate. After several hours of growth, the initially inoculated area saturates and the boundaries of the microcolonies coalesce to form a single large colony. This initial growth phase gives rise to the “homeland” [6] visible as the yellow (i.e. mixed) center of the colonies in Figure 1a. After the homeland is formed, colony development proceeds in the sectored growth-phase in which sectors expand radially from the colony’s perimeter. As the quasi-2D community expands radially over the plate, the population sectors expand or shrink at their (1D or fractal [10]) edges where cells of one type locally compete with their neighbors of another type. Typical sectored patterns of WT and GASP cells growing in a colony inoculated from a 1:1 mixture of WT and GASP bacteria can be seen in Figure 1a. The community only expands at the colony’s perimeter where the competitive interaction can be evaluated by measuring the relative abundance of the two types, while the interior of the colony presents a ‘frozen record’ of the interaction [6]. Such colonies provide an elegant and simple way to study range expansions and spatial competition as they are easy to handle and visualize in the laboratory.

We inoculated solid agar plates (LB medium + 1.5% agar) with droplets having various WT-GASP fractions, and tracked the abundance of both strains during the sectored growth phase (see Figure 1b). Figure 1c shows the time evolution of the GASP fraction of colonies inoculated with starting GASP fractions of 0.1, 0.5, and 0.9. These time traces reveal two points: (1) for the inoculated GASP fractions of 0.1 and 0.5, the GASP fraction has increased when sectored growth commences, (2) the GASP fraction stays fairly constant during sectored growth.

The increased GASP fraction at the onset of sectored growth relative to the inoculated GASP fraction, suggests that the GASP strain has a competitive advantage over the WT when the homeland is formed. In order to test whether the competitive advantage of the GASP strain during formation of the homeland was caused by growth rate differences, we measured the growth rate of pure WT and GASP cultures during exponential growth in liquid batch cultures. The average doubling time of the GASP strain was 45 ± 4 min (mean ± SEM, *n* = 8), whereas the WT strain doubled every 49 ± 6 min (mean ± SEM,* n* = 7). This (marginal) difference is not significant (student’s* t* test), and growth rate differences thus likely do not explain the increase in GASP fraction during formation of the homeland. Previous studies using well-mixed batch cultures [12, 29] have shown that the GASP mutant delays its entry into stationary phase and saturates at higher population densities relative to the WT. It is possible that a similar effect is at play during formation of the homeland, allowing the GASP strain to sustain growth while the WT limits its growth in response to crowding. In the absence of clear growth rate differences, prolonged growth by the GASP strain, and thus higher (saturation) densities, could explain the increased GASP fraction at the onset of sectored growth.

The competitive superiority of the GASP strain during homeland-growth initially translates into an increased GASP fraction, however, when growth proceeds in the sectored phase, the increase in the population share of the GASP strain levels off. Figure 1c shows that in this regime the interaction between the WT and GASP strain is neutral, i.e. the two strains coexist at constant fractions. It is informative to consider the growth state that characterizes the sectored phase. During sectored growth, the colony’s radius grows linearly [9] resulting in an approximately quadratic increase in population size over time. A quadratic accumulation of biomass holds the middle ground between exponential growth (governed by *E. coli*'s housekeeping sigma factor *rpoD*) and stationary-phase growth (regulated by *rpoS*). Furthermore, as the colony expands its territory fresh nutrients are available beyond the colony’s edge, ruling out a scenario of severe nutrient deprivation at the expanding population front. The presence of fresh nutrients, together with a growth phase that is not exponential nor stationary, suggest that the *rpoS* regulon may not be (strongly) induced during sectored growth, rendering the *rpoS* WT and *rpoS819* GASP mutant strains neutral. This hypothesis is in agreement with the observed neutrality of the WT and GASP strain in the sectored growth phase.

**Figure 2:**
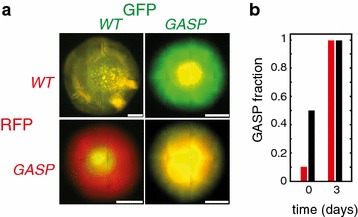
The WT-GASP community on semi-solid agar plates. **a** WT-GASP competition on semi-solid plates (LB + 0.5% agar) inoculated at a 1:1 ratio. On semi-solid agar, *E. coli* engage in collective swarming motility. The swarming edge of colonies inoculated with a 1:1 mixture of WT and GASP cells (*left bottom* and* right top*) consists exclusively of GASP cells, indicating that GASP cells have a large competitive advantage over WT cells on semi-solid agar. Controls, WT/WT (*left top*) and GASP/GASP (*right bottom*), show* yellow* colonies, indicating that the fluorescent reporters are neutral. All* scale bars* indicate 3 mm. **b** GASP fraction at day 0 and 3 for GASP starting fractions of 0.1 (*red bars* $n$ = 2) and 0.5 (*black bars* $n$ = 6). For both initial conditions the GASP strain outcompetes the WT.

We investigated a different setting by inoculating WT and GASP mixtures on semi-solid agar plates (LB medium + 0.5% agar). On semi-solid agar, *E. coli* engage in collective swarming motility. Therefore, in addition to the contribution from growth and division, dispersal mainly results from swarming. Figure 2a shows that GASP *E. coli* have a large competitive advantage over WT when inoculated on semi-solid agar: colonies inoculated with 1:1 WT:GASP mixtures were entirely dominated by GASP cells. Even when the GASP strain initially was a 10% minority, it outcompeted the WT strain entirely by day 3 (see Figure 2b). Microarray studies [35, 36] have shown that genes related to flagellar synthesis are up-regulated in *rpoS* knock-out strains. The overwhelming advantage of the GASP mutant on swarming plates, suggests that the attenuated functioning of its RpoS possibly increases flagellar synthesis and as a result may enhance the ability of GASP mutants to swarm.

## Discussion

Table 1 summarizes the results of previous studies of the WT-GASP community and those presented above. The WT and GASP strain coexist in three types of microfabricated habitats [12, 32, 33] and on solid agar plates, in contrast, the GASP strain outcompetes the WT on semi-solid agar plates and in well-mixed batch cultures. A comparison of the various competitive outcomes thus shows that different spatial constructions can indeed lead to qualitatively distinct community compositions. This observation cautions against the notion of investigating ‘the effect of space’ by contrasting the community dynamics observed in a *single* implementation of spatial structure to observations in a well-mixed culture. Despite the usefulness of such comparisons, our results indicate that the observed dynamics can be particular to the specific experimental implementation of spatial structure.

**Table 1:** The same WT-GASP *E. coli* community in different spatial constructions

Spatial construction	Well-mixed	Semi-solid agar	Solid agar	MHP1	MHP2	MHP3
Strain coexistence?	No	No	Yes	Yes	Yes	Yes
References	[12, 29]	This study	This study	[12]	[32]	[33]

Well-mixed corresponds to shaken flasks and LB medium; semi-solid agar corresponds to plates with LB medium + 0.5% agar; solid agar corresponds to plates with LB medium + 1.5% agar; MHP1 corresponds to a microhabitat with no medium replacement; MHP2 corresponds to a microhabitat consisting of a linear array of 85 coupled chambers in which the left 42 chambers are connected to medium reservoir while the right 43 are not; MHP3, corresponds to a microhabitat consisting of a linear array of 85 coupled chambers in which the odd numbered patches have access to an external medium reservoir while the even numbered patches do not.

**Figure 3:**
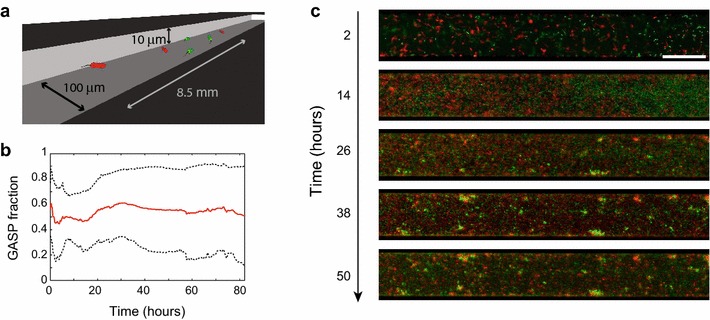
WT and GASP coexist in microfabricated chambers. **a** Sketch of a microfabricated habitat as used in** b**,** c**, and [12]. **b** GASP fraction over time showing the long-term coexistence of both strains co-inhabiting a microhabitat. Mean of eight experiments (*red solid line*),* black dashed lines* indicate the mean ± the standard deviation. Adapted from Ref. [12]. **c** Spatial time series of one experiment where GASP and WT coexist. See Ref. [12] for more data on microhabitat-based coexistence.

Similar competitive outcomes observed in different implementations of spatial structure do not necessarily result from similar underlying dynamics. WT and GASP cells interact neutrally during sectored growth, which we hypothesize stems from a low induction of *rpoS*, rendering the strains neutral. In the microfabricated habitat of Ref. [12] on the other hand, nutrients are limiting and the WT-GASP community reaches the habitat’s carrying capacity resulting in stationary-phase conditions and consequently induction of *rpoS*—still, coexistence was observed in Ref. [12] (see Figure 3). This coexistence was suggested to arise from the dynamic self-structuring of the WT-GASP community: although clusters of GASP cells were outcompeting WT cells locally (and vice versa), cellular dispersal allowed planktonic cells of both strains to establish new subpopulations in unoccupied territory facilitating the co-occurrence of both strains at the global scale. The coexistence observed in two additional types of microfabricated habitats (Refs. [32, 33]) was attributed to the two strains spatially separating due to spatial differences in habitat quality (such heterogeneities were not present in the habitats used in Ref. [12]).

Also in the two spatial constructions where the GASP strain drives the WT strain extinct (well-mixed flasks and semi-solid plates) different mechanisms are responsible for the GASP strain’s superiority. In well-mixed flasks GASP cells outcompete WT cells by delaying their entry into stationary phase, whereas differences in swarming ability are the likely the cause of GASP superiority on semi-solid plates.

Radially expanding colonies and microhabitats both restrict cell–cell interactions to relatively small scales (when compared to a well-mixed flask), yet they differ in important respects. On solid agar, *E. coli* cells are immotile and dispersal in colonies results from growth and division only. The microhabitats used in Refs. [12, 32, 33] on the other hand, consist of micro-scale patches filled with liquid medium that support swimming motility. Bacteria inhabiting a microhabitat can thus be sessile (surface associated) or planktonic (free swimming) and switch between those modes, allowing cells to aggregate in biofilms and disperse from those dynamically (see Figure 3). Such dynamic self-structuring of the community in space and time is absent in colonies growing on solid agar. Other differences exist between the various implementations of spatial structure considered here. The availability of resources for instance, was varied in the three studies that used microhabitats: in Refs. [32, 33], the bacteria inhabited chambers that were connected to external nutrient reservoirs. In Ref. [12] and Figure 3 such external resource supply is absent (see Table 1). The situation of colony growth on an agar plate is reminiscent of the microhabitats with an external nutrient supply, as a source of fresh nutrients extends beyond the colony edge.

## Conclusion

The observations presented here, and in previous work [12, 32, 33], emphasize the idiosyncrasy of spatial community dynamics: different implementations of space achieved by different culturing methods may not only lead to quantitatively different communities (changes in the relative abundance of types) but can also lead to qualitatively different outcomes of the long-term community dynamics (coexistence versus extinction and loss of biodiversity). Conversely, similar outcomes in different habitats could be the result of distinct competitive interactions. Although this work focused on one particular set of strains, we expect similar implications to hold in other microbial systems. It is therefore important to keep these subtleties in mind when evaluating the long-term community structure resulting from spatial competition across different laboratory constructions representing the spatial ecology of microbes.

## Methods

### Strains and growth conditions

All strains used in this study have been described before in Refs. [12, 32]. Wild-type strains JEK1036 (green) and JEK1037 (red) are *Escherichia coli* W3110 carrying the WT *rpoS* allele, and are lacYZ::GFPmut2 and lacYZ::mRFP, respectively. GASP mutants JEK1032 (green) and JEK1033 (red) have the GASP phenotype and carry the *rpoS819* allele (described in Ref. [28]) and the same fluorescent markers and genetic background as strains JEK1036 and JEK1037, respectively. Prior to all experiments cells were taken from a –80°C glycerol stock and grown overnight (30°C, 200 rpm) in lysogeny broth (LB).

Growth rates of pure WT and GASP cultures were obtained by measuring the absorbance of 200 μL cultures growing at 30°C, shaken at 600 rpm in a 96 well plate using a BMG FluoStar Optima plate reader. Growth curves were log-transformed and fitted with a first-order polynomial in Matlab to obtain doubling times.

### Plate assays

The four *E. coli* strains (JEK1032, JEK1033, JEK1036 and JEK1037) were grown overnight in liquid cultures (30°C, shaken at 200 rpm) and separately diluted 1/500 in LB medium containing 100 μM IPTG and grown to OD_600_ = 0.25. LB agar plates (1.5% agar for solid plates, 0.5% agar for semi-solid plates; supplemented with 100 μM IPTG) were inoculated with 1 μL of WT-GASP mixtures (GASP fractions of: 0.1, 0.5 and 0.9) and incubated at 30°C for 72 h. Colonies were imaged using an inverted Olympus IX81 microscope equipped with a 10× (NA = 0.3) objective, an ORCA-R2 camera (Hamamatsu) and a motorized stage controlled using μManager software [37]. The sample was illuminated using an X-cite 120 Q light source (Lumen dynamics).

Images were background corrected and converted to polar coordinates using the center of the homeland as the origin through a custom Matlab script (see Figure 1b). The converted image was used to evaluate the GASP fraction over time during the sectored growth phase. A previous study [9] demonstrated that the radius of *E. coli* colonies expands linearly over time, we therefore mapped the obtained fraction onto a linear time axis.
